# Effects of dwarfing allele *sd1-d* originating from ‘Dee-geo-woo-gen’ and its tall alleles *SD1-in* and *SD1-ja* on morphological characteristics concerning dry-matter production and photosynthesis on the genetic background of *indica*-rice IR36

**DOI:** 10.1270/jsbbs.22016

**Published:** 2022-10-21

**Authors:** Misa Kamimukai, Birendra Bahadur Rana, Mukunda Bhattarai, Masayuki Murai

**Affiliations:** 1 Faculty of Agriculture and Marine Science, Kochi University, 200 Otsu, Monobe, Nankoku, Kochi 783-8502, Japan; 2 Nepal Agriculture Research Council (NARC), Khumaltar, Lalitpur, Kathmandu, Nepal; 3 National Agricultural Genetic Resource Center (Genebank), Nepal Agriculture Research Council (NARC), Khumltar, Lalitpur, Kathmandu, Nepal; 4 Emeritus Professor of Kochi University, 201 Higashino, Noichi-cho, Konan, Kochi 781-5213, Japan

**Keywords:** *Oryza sativa*, dwarfing gene, *sd1*, yield, dry matter production, harvest index, translocation of carbohydrates

## Abstract

*sd1-d* has been utilized to develop short-culmed *indica* varieties adaptable to higher fertilizer-applications. Its tall alleles *SD1-in* and *SD1-ja* are harbored in *indica* and *japonica* subspecies, respectively. *SD1-in* possesses a higher effect on elongating culm than *SD1-ja*. The *sd1-d* of *indica* IR36 was substituted with *SD1-in* or *SD1-ja* through recurrent backcrossing with IR36, and two tall isogenic lines (“5867-36” and “Koshi-36”) were developed. IR36, 5867-36 and Koshi-36 were grown in a paddy field, and the effects of *sd1-d*, *SD1-in* and *SD1-ja* on morphological characteristics concerning dry-matter production and photosynthesis were compared mutually. *sd1-d* diminished dry weight of total brown rice/m^2^ and total dry matter weights, but enhanced harvest indexes, compared with *SD1-in*. In IR36, shorter lengths of the first (flag) to third leaves, and more panicle-bearing stems, caused by *sd1-d*, compared with *SD1-in*-carrying 5867-36, and erect first leaves, not caused by *sd1-d*, could construct the canopy structure appropriate for obtaining a high rate of photosynthesis at an optimum LAI. Koshi-36 could be used for a mid-mother line to develop *indica* varieties adaptable to middle and low fertilizer-applications, due to higher effect of *SD1-ja* on yielding ability, compared with that of *sd1-d*, no breaking-type lodging, and resistances to diseases and pests.

## Introduction

The *sd1-d* allele originating from ‘Dee-geo-woo-gen’ at the *sd1* locus on chromosome 1 has been intensively used to develop high-yielding *indica* varieties adaptable to high fertilizer-applications, such as IR8, IR36 and IR72 (Aquino and Jennings 1966, De Datta *et al.* 1968, Murai and Yamamoto 2001, Murai *et al.* 2003, Peng *et al.* 1999).

The wild-type allele *SD1* encodes the gibberellin biosynthetic enzyme GA20 oxidase (*GA20ox-2*) that catalyzes late steps of gibberellin biosynthesis, while *sd1-d* includes the deletion of 383 bp between the two sites of exon 1 and exon 2, resulting in the loss of the enzymic function (Ashikari *et al.* 2002, Monna *et al.* 2002, Sasaki *et al.* 2002, Spielmeyer *et al.* 2002). The dominant allele *SD1* at the locus is differentiated into *SD1-in* and *SD1-ja* which are harbored in *indica* and *japonica* subspecies, respectively (Murai *et al.* 2011). The effect of elongating culm is higher in *SD1-in* than in *SD1-ja*, which can be one cause of the inter-subspecific difference in height (Murai *et al.* 2011). Nonsynonymous single-nucleotide polymorphisms between *SD1-in* and *SD1-ja* were detected at the two sites in exon 1 and exon 3 of the *sd1* locus (Asano *et al.* 2011, Murai *et al.* 2011).

The *sd1-d* of *indica* IR36 was substituted with *SD1-in* or *SD1-ja* through the recurrent backcrossing with IR36, and two tall isogenic lines carrying the respective tall alleles were developed (Murai *et al.* 2011). In our previous study (Rana *et al.* 2021), field tests were performed for the two tall isogenic lines and IR36 in the three years, and we obtained the results as follows: *SD1-in* decreased panicle number per m^2^ but increased spikelet number per panicle and 1000-grain weight, compared with *sd1-d*, resulting in the increase of yield; and *SD1-ja* did not significantly affect yield, compared with *sd1-d*, mainly because the decrease of panicle number per m^2^ was compensated by the increase of 1000-grain weight. The increases in 1000-grain weight by *SD1-in* and *SD1-ja* were due to the increases in both the length and width of lemma, and the increase in the length alone of it, respectively.

Donald and Hamblin (1976) demonstrated that grain yield can be decomposed into biological yield and harvest index in cereal crops, and the latter trait can be a criterion for selecting a genotype adaptable to cultivated conditions with high planting-density and/or high fertilizer-application. The former trait could be approximated with total plant weight at maturity in rice. Tanaka *et al.* (1966) reported that harvest index was negatively correlated with height at maturity among rice varieties with high, middle and low heights. According to Murai *et al.* (2002a), Taichung 65, which carries *SD1-ja* (Rana *et al.* 2021), was significantly higher in total plant dry-weight per m^2^ at maturity than its *sd1-d* isogenic line, while the former was rather lower in harvest index than the latter. In the present study, total plant dry-weight per m^2^ at maturity, and harvest index were measured for the materials of the two tall isogenic lines and IR36 grown in the three years (Rana *et al.* 2021). Additionally, total plant dry-weight per m^2^ at 80%-heading was measured. From this trait and that at maturity, increase of total plant dry-weight after heading was calculated for the lines-variety in the three experimental years, which principally contributes to grain yield (Tanaka *et al.* 1966, 1968). Seo and Chamura (1979), and Weng *et al.* (1982) suggested that carbohydrates (mainly sugars and starch) stored in leaf sheaths and culms before heading contributed to yield more or less in rice varieties. The amount of carbohydrates transferred from leaf sheaths and culms to panicles during maturing stage can be estimated by the difference on dry-matter basis between total brown rice weight and increase of total plant weight after heading (Amano *et al.* 1993). This trait was calculated by combining the data of the concerned traits of the lines-variety in the three experimental years, in order to examine whether the three alleles at the *sd1* locus affect this trait or not.

The lengths of the first (flag) to third leaf blades, LAI (leaf area index) and angle of the first leaf were measured for the lines-variety in the three or two of the three experimental years; because these characteristics are closely related to the light-receiving canopy structure of a plant community (Tanaka 1972).

On the basis of the results obtained, the effects of *SD1-in*, *SD1-ja* and *sd1-d* on each of total dry weight at maturity, harvest index, increase of total plant weight after heading, amount of translocation, morphological characteristics of the leaf blades, and other traits are compared with each other, on the common genetic background of IR36. Moreover, we discuss how *sd1-d* changes the canopy structure of plant community that is closely associated with the efficiency of photosynthesis, compared with *SD1-in*. From another view point, the utility of *SD1-ja*, and that of the *SD1-ja* isogenic line of IR36 itself as a mid-mother line for developing new *indica* varieties are discussed.

## Materials and Methods

### Tall isogenic lines

The two tall isogenic lines possessing *SD1-in* and *SD1-ja*, denoted by “5867-36” and “Koshi-36”, respectively, were developed after 17 backcrosses with IR36, using IR5867 and ‘Koshihikari’ as their donors (Murai *et al.* 2011, Rana *et al.* 2021).

### Cultivation in experimental field

The two tall isogenic lines and IR36 were grown by the same way of cultivation in 2017, 2018 and 2019 (Rana *et al.* 2021), as summarized as follows. Twenty-day seedlings were transplanted at a spacing of 30cm × 15cm (22.2 hills/m^2^) with two seedlings per hill to a paddy field of Faculty of Agriculture and Marine Science, Kochi University, Nankoku, Japan, on May 3. The total amount of chemical fertilizer applied to the paddy field by basal dressing and top-dressing was at the rate of 8.00, 6.86 and 7.62 g/m^2^ for N, P_2_O_5_ and K_2_O, respectively. For the two tall isogenic lines and IR36, the randomized block design with three replications was employed. Each plot comprised 29 hills × 6 rows (174 hills).

Regarding 80%-heading date, both 5867-36 and Koshi-36 were 27th or 28th of July, and IR36 was 29th or 30th of July, in the three experimental years (Rana *et al.* 2021).

### Measurements of dry matter, leaf area, and harvest indexes

At each of 80%-heading stage and maturity in each line/variety, panicle number per hill was counted for nine adjacent hills in each plot. Five hills, which had intermediate panicle numbers per hill, were selected and sampled from the nine hills at each of the two stages. At each stage, total dry weight excluding roots of each hill was measured after drying at 75°C for two days; simultaneously, its panicles were cut out and weighed. At 80%-heading stage, the total area of living leaf blades in each hill and its dry weight were measured.

Two harvest indexes for total brown rice and brown rice with thickness ≥ 1.5 mm, on dry-matter basis, were estimated as follows: 1) the percentage of panicle dry weight to total (plant) dry weight at maturity (a) was calculated; 2) the percentage of either dry weight of total brown rice or that of brown rice with thickness ≥ 1.5 mm to the dry weight of the panicles (b) was estimated, using the moisture content of brown rice (approximately 12%, after drying with a drying oven), the oven-dried weights of the two kinds of brown rice and the oven-dried panicle weight (Rana *et al.* 2021), and the ratio of panicle dry weight to oven-dried panicle weight; 3) an estimated value of each of the two harvest indexes (%) for total brown rice and brown rice with thickness ≥ 1.5 mm was obtained by (a × b)/100.

### Measurements of lengths and widths of leaf blades

The lengths and widths of the first (flag) to third leaf blades (hereafter “1st, 2nd and 3rd leaves”) in the highest culm in each of ten hills of each plot were measured for the two tall isogenic lines and IR36, about 20 days after 80%-heading stage.

### Increase of total dry weight after heading; and Amount of translocation

Increase of total dry weight after heading (ΔW) was calculated from the difference between the total dry weight at 80%-heading and that at maturity. The amount of translocated carbohydrates from culms and leaf sheaths to panicles (“amount of translocation”) was estimated by the difference between dry the weight of total brown rice and ΔW, according to Amano *et al.* (1993).

### ANOVA (Analysis of variance)

For each of the traits mentioned above, ANOVA was performed according to the completely randomized design, assuming the three replications in each of the three lines-variety in each of the three experimental years as the source of three randomized data for error variance, and the lines-variety and the years as the two factors based on the fixed effect model.

### Measurements of 1st (flag)-leaf angles during maturing stage

Angle between a 1st leaf blade and the culm (hereafter “1st-leaf angle”) was measured by placing the center of a protractor (radius = 4.3 cm) at the auricle of the 1st leaf in the highest culm of each of 10 hills per plot of each line/variety at 5, 15 and 25 days after 80%-heading and at maturity in 2017 and 2018. ANOVA was conducted by a way similar to that mentioned above, using the two-year data of the three lines-variety at each of the four measuring times after heading. In addition, one way ANOVA was performed to examine the temporal change in 1st-leaf angle after heading within each of the three lines-variety, assuming the three replications at each of the four measuring times in each of the two experimental years as the source of three randomized data for error variance, and the combined data of the four measuring times and the two years as the single factor.

## Results

### Total dry weight and harvest index

Table 1 shows the results of ANOVA for all of the traits examined in 5867-36, Koshi-36 and IR36 in 2017, 2018 and 2019. Regarding total dry weight per m^2^ at 80%-heading, that at maturity, harvest index of total brown rice yield, harvest index of brown rice with thickness ≥ 1.5 mm, the effects of both the lines-variety and the years were statistically significant, but the interaction between them was not significantly effective. Table 2 shows the performances of the lines-variety in the traits mentioned above in the three experimental years. Regarding total dry weight at 80%-heading as well as that at maturity, statistically significant differences were not noticed between Koshi-36 and IR36 in each of the three years. In the average of the three years, Koshi-36 was 4 or 5% higher than IR36 in these two traits, although being not significantly different statistically. 5867-36 was higher than IR36, regarding both total dry weight at 80%-heading and that at maturity in the three years, although the differences in 2017 were not statistically significant. In the average of the three years, 5867-36 was significantly higher by 16% than IR36, identically in the two traits. Among the three years within each of the lines-variety, each of the two traits was lower in 2018 than in the other two years, although differences were not statistically significant in the most of the cases. It is inferred that the decreases of the two traits in 2018 in each line/variety are due to the decrease of panicle number per m^2^ in 2018 in each line/variety (Rana *et al.* 2021).

Regarding harvest index of total brown rice (Table 2), the lines-variety were in the order IR36 ≥ or > Koshi-36 ≥ or > 5867-36 in the three experimental years, where “≥” indicates that the former is higher than the latter but being not statistically significant at the 5% level of probability, and “>” indicates significant difference. Furthermore, the average of the three years was in the order IR36 > Koshi-36 > 5867-36. This trait was tightly correlated with harvest index of brown rice with thickness ≥ 1.5 mm among the nine combinations of the three lines-variety and the three experimental years (r = 0.988, significant at the 1% level). The former trait was higher by 0.6 to 1.4% than the latter trait in the nine combinations of the lines-variety and the years.

### Dry weight of total brown rice, increase of total dry weight after heading, and amount of translocation

For dry weight of total brown rice, one of the indicators of yield, the effects of both the lines-variety and the years were statistically significant, but the interaction between them was not significantly effective (Table 1). In this trait, the lines-variety were in the order IR36 ≥ 5867-36 ≥ Koshi-36 in 2017, and were in the order 5867-36 ≥ Koshi-36 ≥ IR36 (5867-36 > IR36) in each of 2018 and 2019 (Table 3). The order was 5867-36 > Koshi-36 ≥ IR36 in the average of the three years. Among the nine combinations of the lines-variety and the years, this trait was tightly correlated with brown rice yield with thickness ≥ 1.5 mm containing 15% moisture (Rana *et al.* 2021) (r = 0.995, significant at the 1% level). Among the three years within each of the lines-variety, this trait was lower in 2018 than in the other two years, although differences were not statistically significant in most of the cases. It is inferred that the decrease of this trait in 2018 within each line/variety was due to the decrease of panicle number per m^2^ in 2018 (Rana *et al.* 2021).

The effects of the lines-variety, the years and the interaction between them were not significantly effective in increase of total dry weight after heading, amount of translocation, and contribution of translocation to dry weight of total brown rice (%) (Table 1). In each of the three traits, significant differences were not noticed not only in each of the three experimental years but also in the average of the three years (Table 3). Nevertheless, a considerable difference (48 g/m^2^, 14%) was noticed between 5867-36 and IR36 on the average of the three years regarding increase of total dry weight after heading, although the difference was not statistically significant due to the relatively large LSD_(5%)_ (68 g/m^2^) compared with the LSD_(5%)_ in dry weight of total brown rice (24 g/m^2^). Besides, it is noteworthy that amount of translocation contributed considerably to dry weight of total brown rice: the percentages on the average of the three years were 23.1 to 27.9% in the lines-variety.

### Lengths and widths of leaf blades, and LAI

Before comparing the three lines-variety mutually, the morphological characteristics of 1st to 3rd leaves were examined. The 1st leaf was shorter by 13.4 to 19.9 cm than 2nd leaf in the 9 combinations of the lines-variety and the years (Table 4). The 2nd leaf was shorter by 1.3 to 4.5 cm than 3rd leaf in 7 of the 9 combinations, while 2nd leaf was longer by 2.1 and 2.4 cm than 3rd leaf in 5868-36 and IR36, respectively, in 2018. On the other hand, leaf width decreased from 1st to 3rd leaves, step by step, in each of the lines-variety in each of the years.

As shown in Table 1, the effects of the lines-variety and the years were significant in the lengths of 1st and 2nd leaves. The effect of lines-variety alone was significant in 3rd leaf length, and the widths of 2nd and 3rd leaves. The effect of the years alone was significant in 1st leaf width, LAI and the estimated LAI. The interaction between the two factors was not significantly effective for all of the traits mentioned above. Regarding 1st leaf length, Koshi-36 was not significantly different from IR36 in the three years. 5867-36 was longer than the other two line-variety in this trait in the three years, although being not significantly different statistically in 2017. This trait was in the order 5867-36 > Koshi-36 ≥ IR36 in the average of the three years. Regarding each of 2nd leaf length and 3rd leaf length, the lines-variety were in the order 5867-36 > Koshi-36 > IR36 in each of the three years. In 2nd leaf length, 5867-36 and Koshi-36 were longer by 29 and 11%, respectively, than IR36 in the average of the three years. In 3rd leaf length, 5867-36 and Koshi-36 were longer by 28 and 19%, respectively, than IR36 in the average of the three years. The 1st leaf as well as 2nd leaf was longer in 2018 than in the other two years, with the exception of the 1st leaf of Koshi-36. However, such yearly differences were not noticed in 3rd leaf.

Regarding 1st leaf width, the lines-variety were in the order 5867-36 ≥ Koshi-36 ≥ IR36 (5867-36 > IR36) in the average of the three years (Table 4), although the effect of the lines-variety was not significant (Table 1). This trait was higher in 2018 than in the other two years in each of 5867-36 and Koshi-36, while such a yearly difference was not noticed in IR36. Regarding each of 2nd leaf width and 3rd leaf width, the lines-variety were in the order IR36 > 5867-36 ≥ Koshi-36 in the average of the three years.

In each of 1st, 2nd and 3rd leaves, the variation among the lines-variety in leaf width was smaller than that in leaf length, in each of the three years (Table 4).

In terms of LAI, there were not significant differences among the lines-variety in each of the years as well as the average of the three years. In IR36, this trait was significantly higher in 2017 than in 2018, but such yearly differences were not noticed in 5867-36 and Koshi-36. From the data mentioned above and Rana *et al.* (2021), the estimated LAI (m^2^/m^2^) was calculated as follows: the length × width values from 1st to 3rd leaves in a culm were cumulated, and this cumulated value was multiplied by panicle number per m^2^. The lines-variety were not significantly different from each other in the average of the three years in this trait. The regression equation of y = 0.612x + 1.251 was obtained, where x and y are the estimated values and real measurements of LAI, respectively (r = 0.686, significant at the 5% level). Hence, an estimation of LAI at heading could be obtained, to some extent, from the lengths and widths of 1st to 3rd leaves, and panicle number per m^2^.

### 1st-leaf angles during maturing stage

The effects of both the lines-variety and the years were statistically significant in all of the 1st-leaf angles at 5, 15 and 25 days after 80%-heading, and maturity (Table 5). The interaction between them was significantly effective in the 1st-leaf angles at 5 and 15 days after 80%-heading. Regarding 1st-leaf angle, Koshi-36 was not significantly different from IR36 through the four measuring times during maturing stage in both years. 5867-36 was significantly larger than IR36 at 15 and 25 days after 80%-heading in 2017, and at 5 days after 80%-heading in 2018, in which the largest difference was 8.1 degrees angle; whereas significant differences between 5867-36 and IR36 were not noticed in the other five combinations of the measuring times and the years. Within each of the lines-variety, temporal change in 1st-leaf angle during maturing stage (Table 6) was examined, as follows. In 2017, 1st-leaf angle was the smallest at 5 days after 80%-heading, in each line/variety, while differences were not significant or significant but small among the other three measuring times. In 2018, little temporal changes in this trait were noticed through the four measuring times in each line/variety.

## Discussion

Short-culmed varieties with erect leaves are advantageous for pursuing high yield, not only in the Philippines but also in Hokkaido, one of the northernmost rice-growing areas in the world (Tanaka *et al.* 1966, 1968). According to Murai *et al.* (1983), and Murai and Kinoshita (2003), the high-yielding and short-culmed varieties of Hokkaido had more erect flag leaves at maturity (62.4 degrees angle on average, n = 11) than the low-yielding and long-culmed varieties of there, comprising indigenous varieties and pure-line selections derived from some of them (99.3 degrees angle on average, n = 9). In the present study, the differences in this trait between Koshi-36 and IR36 were little during maturing stage in the two years, and the differences between 5867-36 and IR36 were either not significant or significant but 8.1 degrees angle at maximum (Table 6). Hence, it is inferred that *sd1-d* almost does not affect 1st-leaf angle, compared with *SD1-in* as well as *SD1-ja*. In this trait, IR36 was 4.2 degrees angle at maturity in the average of the two years. This value of the trait was even narrower than the 30 degrees angle of ‘Narukaze’, the most erect-leafed variety of those examined by Murai *et al.* (1983). Therefore, it seems that the characteristic of the erect 1st leaf of IR36 was obtained through the process of breeding but is not due to the effect of *sd1-d*.

Tanaka (1972) demonstrated that the efficiency of photosynthesis can be higher in the canopy of rice plants with short, erect and many leaves than in that with long, drooping and few leaves. In the present study, *sd1-d* decreased 1st, 2nd and 3rd leaf lengths by 18, 23 and 22%, and by 2, 10 and 16%, compared with *SD1-in* and *SD1-ja*, respectively, in the average of the three years (Table 4). On the other hand, *sd1-d* increased panicle number per m^2^ by 18 and 5%, compared with *SD1-in* and *SD1-ja*, respectively, in the average of the three years (Rana *et al.* 2021). Accordingly, it seems that *sd1-d* involves the effect on improving canopy structure to enhance efficiency of photosynthesis, compared with *SD1-in*. On the other hand, *sd1-d* may not drastically affect canopy structure, compared with *SD1-ja*; because the reduction percentages by *sd1-d* to *SD1-ja* in the three leaf lengths were relatively low, and particularly, the reduction (2%) was not statistically significant in the length of 1st leaf which first intercepts sunlight from the leaves below it.

According to Tanaka *et al.* (1966, 1968), higher-yielding varieties had higher performances regarding increase of total dry weight after heading, in the Philippines as well as in Hokkaido. In the present study, this trait mainly contributed to dry weight of total brown rice (≒ yield) in the three lines-variety in the three experimental years (Table 3). 5867-36 was rather higher in this trait than IR36 in the average of the three years. In 5867-36, mutual shading in its canopies was not seriously intensive, because its LAIs at 80%-heading (3.25 to 3.65, Table 4) were lower than the optimum LAI of 6 proposed by Tanaka (1972), and its 1st leaves were erect during maturing stage (Table 6). This situation of 5867-36’s plants (Tables 4, 6) seems to have brought about the rather higher performance in increase of total dry weight after heading and the higher dry weight of total brown rice in 5867-36 than in IR36 (Table 3), resulting from its rather larger sink size and higher fertilized-spikelet percentage than those of IR36 (Rana *et al.* 2021).

Hybrid-rice varieties achieved brown-rice yields of about 1000 g/m^2^ or higher in China, in which the contribution of amount of translocation to total brown-rice yield was 38% at maximum (Amano *et al.* 1993, 1996a, 1996b). It is known that *Ur1* gene on chromosome 6 increases spikelet number per panicle, and is able to enhance brown-rice yield by enlarging sink size (Murai *et al.* 2002b). Kamimukai *et al.* (2020) reported that a *japonica Ur1*-carrying line with the characteristic of extremely late heading had the total brown rice yield of 766 g/m^2^, in which the contribution of amount of translocation to the yield was 34%. The contributions of amount of translocation were 23.1, 24.7 and 27.9% for 5867-36, Koshi-36 and IR36, respectively, on the average of the three years, in which the mutual differences were not statistically significant (Table 3). According to a yield test performed under an optimum cultivated condition (total nitrogen-application = 18 g/m^2^, and early cultivation) at the Shikoku National Agricultural Experiment Station in Kagawa Prefecture, Japan (Komatsu *et al.* 1984), the brown-rice yield of IR36 (773 g/m^2^) was intermediate between that of the highest-yielding *indica*-type variety Suweon 258 (947 g/m^2^) and that of a representative Japanese *japonica* variety ‘Nipponbare’ (646 g/m^2^), probably reflecting its yielding ability. Hence, *sd1-d* may not possess a significant effect on amount of translocation, compared with *SD1-in* and *SD1-ja*, on the genetic background of IR36 with the fairly high yielding-ability.

Among the nine combinations of the lines-variety and the years, total dry weight at maturity was tightly correlated with that at 80%-heading, and both the former and latter traits were highly correlated with dry weight of total brown rice (r’s = 0.963, 0.877 and 0.871, respectively, all significant at the 1% level of probability; Tables 2, 3). However, harvest index of total brown rice as well as that of brown rice with thickness ≥ 1.5 mm was in the order IR36 > Koshi-36 > 5867-36 in the average of the three years (Table 2). Therefore, the higher yielding-ability of *SD-in*-carrying 5867-36, compared with *sd1-d*-carrying IR36, was due to the increase of biomass production but not due to the enhance of harvest index. According to Tanaka *et al.* (1964), three long-culmed *indica* varieties including a representative variety ‘Peta’ were higher-yielding in a cultivated condition with no nitrogen-application than in that with the total nitrogen-application level of 10 g/m^2^. According to Tanaka *et al.* (1966) and Kaneda (1975), long-culmed *indica* varieties, presumably carrying *SD1-in*, had been broadly grown with both no or limited application of chemical fertilizers and lack of irrigation equipment in tropical and subtropical regions of Asia, before the release of IR8 in 1966. Hence, the higher potential yielding-ability by *SD1-in*, compared with that by *sd1-d*, may confer an advantage on such long-culmed *indica* varieties adaptable to cultivated conditions without applying chemical fertilizers.

Nevertheless, serious lodging was observed in 5867-36 at the late stage of maturing in the three experimental years, due to the long culm of more than 1 m (Rana *et al.* 2021), and higher total weight of panicle, leaves and internodes above the fifth internode, and lower breaking strengths at the fourth and fifth internodes, compared with those of IR36 (Murai unpublished). Tanaka *et al.* (1964) observed lodging from heading stage in the three long-culmed varieties. Accordingly, it is expected that nitrogen applications still higher than that in the present study (8.00 g/m^2^ in total) would cause destructive lodging after heading in *SD-in*-carrying 5867-36. On the other hand, no lodging was observed in IR36 at maturity in the three experimental years. From the results of research conducted at International Rice Research Institute (IRRI) and another institution in the Philippines (Peng *et al.* 2000), the average values of yield and LAI in IR36 are calculated as 665 g/m^2^ and 5.52, respectively, by assuming that 80% of rough (unhulled) rice is yield of brown rice. In average of the three experimental years, IR36 was 533 g/m^2^ (yield of brown rice with thickness ≥ 1.5 mm) and 3.41 (LAI) (Rana *et al.* 2021, Table 4). These differences in the two traits in IR36 may be principally due to the difference in total nitrogen-application level, viz. 20 and 8 g/m^2^ in the former and latter studies, respectively. Thus, the higher yields with the higher LAIs were obtained by applying the high level of nitrogen in IR36.

In a yield test in a wet season at IRRI (Tanaka *et al.* 1964), two Taiwanese *japonica* varieties, so-called “Ponlai” varieties, had higher yields than both the three long-culmed *indica* varieties and the six Japanese *japonica* varieties at each of the two total nitrogen-application levels of 0 and 10 g/m^2^. Another Ponlai variety Taichung 65 and a *tropical* *japonica* (*javanica*) variety ‘Silewah’ are known to harbor *SD1-ja* (Murai *et al.* 2011, Rana *et al.* 2021). Accordingly, the above experimental result implies that some of the *SD1-ja*-carrying varieties might be adaptable to cultivated conditions at low and middle levels of nitrogen application in the subtropics. According to Yazawa (1975), it was more appropriate to obtain a reasonably high yield with a minimum amount of chemical fertilizer(s) than to pursue high yields by highly nitrogen-responsive varieties like IR8, in the areas of tropical and subtropical Asian countries where chemical fertilizers were costly. For example, rice varieties are recommended to be grown with the total nitrogen-application level of 12 g/m^2^, together with P and K elements, in the subtropical area of Nepal; although the level of application varies from 0 with compost to about 15 g/m^2^ in the country (Baral *et al.* 2019). Yazawa’s suggestion (1975) is consistent with the environment-friendly rice-cultivations which is emphasized recently. For such a purpose, *SD1-ja*-carrying *indica* varieties could be taken into consideration; because Koshi-36 was similar to or rather higher than IR36 in terms of dry matter weight of total brown rice, and total dry matter weights at both 80%-heading and maturity (Tables 2, 3), and non-serious bending without breaking-type lodging, by applying the middle level of nitrogen. This proposal concerning *SD1-ja* is supported by the experimental result that *SD1-ja*-carrying Taichung 65 was higher in yield than its *sd1-d* isogenic line not only at the total nitrogen-application levels of 8 and 12 g/m^2^ but also at that of 4 g/m^2^ (Murai *et al.* 2002a). Peng *et al.* (2000) listed the major improved characteristics of IR36 as follows: short growth duration; good grain quality; and the multiple resistances to brown planthopper, green leafhopper, tungro, blast and bacterial blight. It is postulated that both of Koshi-36 and 5867-36 inherited the major improved characteristics from IR36 in the process of 17 backcrosses. However, it is not easy to introduce a gene/allele from a *japonica* variety into an *indica* variety, because the hybrid sterility and hybrid lethality between *japonica* and *indica* cause reproductive isolation (Oka 1954, 1957, Oka and Doida 1962). Therefore, there is a possibility that Koshi-36 will be a mid-mother line to develop new *indica* varieties adaptable to middle or low levels of nitrogen application.

The effects of *sd1-d* on the morphological characteristics concerning dry matter production and photosynthesis, compared with those of *SD1-in*, are summarized as follows. *sd1-d* diminished dry weight of total brown rice, and total dry matter weights at both 80%-heading and maturity. On the other hand, *sd1-d* enhanced harvest index, suggesting that *sd1-d* confers an advantage in the adaptability to high levels of nitrogen application in IR36. Shorter lengths of 1st leaf and the two leaves below it, and more panicle-bearing stems, caused by *sd1-d*, and erect flag leaves, not caused by *sd1-d*, could construct the light-receiving canopy structure appropriate for obtaining a high rate of photosynthesis at an optimum LAI, such as 6 by Tanaka (1972), in IR36. This characteristic and the high lodging resistance, principally caused by *sd1-d*, seems to enable high yields by applying high levels of nitrogen element in IR36. From another view point, it is suggested that Koshi-36 could be recommended as a mid-mother line to develop new *indica* varieties adaptable to middle or rather low levels of nitrogen application, due to the following reasons: the potential yielding ability of *SD1-ja* higher than or similar to that of *sd1-d*, and the minor degree of bending without breaking-type lodging, principally caused by *SD1-ja*; and the short growth duration, good grain quality, and the multiple resistances to the diseases and pests presumably inherited from IR36 (Peng *et al.* 2000).

## Author Contribution Statement

MK and BBR performed the whole of the experiments and analyses. MB performed the whole of the experiments. MM developed the two tall isogenic lines of IR36, designed and performed the whole of the experiments, and wrote the manuscript.

## Figures and Tables

**Table 1.** ANOVA for all traits measured for 5867-36, Koshi-36 and IR36 from 2017 to 2019, in which the results are shown by F-values

Traits	Lines-variety (L)	Years (Y)	Interaction (L × Y)
Total dry weight at heading (g/m^2^)	7.41**	6.72**	<1
Total dry weight at maturity (g/m^2^)	12.85**	6.46**	<1
Harvest index of total brown rice (%)	26.12**	5.01*	1.51
Harvest index of brown rice with thickness ≥ 1.5 mm (%)	29.37**	9.80**	1.54
Dry weight of total brown rice ^1)^ (g/m^2^) (a)	6.76**	5.75*	1.49
Increase of total dry weight after heading (g/m^2^) (b)	1.07	0.82	<1
Amount of translocation (g/m^2^) (c)	<1	2.43	<1
Contribution of translocation (c/a, %)	<1	1.79	<1
1st leaf length (cm)	22.15**	11.66**	2.77
2nd leaf length (cm)	96.78**	16.85**	3.34
3rd leaf length (cm)	187.33**	3.22	2.44
1st leaf width (cm)	2.83	9.09**	4.42
2nd leaf width (cm)	6.30**	3.05	3.90
3rd leaf width (cm)	11.24**	<1	<1
LAI	<1	3.98*	<1
Estimated LAI ^2)^	1.41	15.50**	2.41

Degrees of freedom for the lines-variety, the years, the interaction and the error are 2, 2, 4 and 18, respectively. *, ** Significant at the 5 and 1% levels of probability, respectively. Note: c = a – b. b = ΔW. ^1)^ Estimated from the total brown rice yield containing 15% moisture (Rana *et al.* 2021). ^2)^ The length × width values from 1st to 3rd leaves in a culm were cumulated, and this cumulated value (m^2^) was multiplied by panicle number per m^2^.

**Table 2.** Total dry weights at 80%-heading and maturity, and two harvest indexes in 5867-36, Koshi-36 and IR36 from 2017 to 2019

Traits	Years	5867-36		Koshi-36		IR36	LSD (5%)
Total dry weight at 80%-heading (g/m^2^)	2017	1054 a	(111)	943 abc	(99)	951 abc	137
	2018	935 abc	(122)	838 cd	(110)	765 d	
	2019	1018 ab	(117)	908 bc	(104)	873 cd	
	Average	1002 a	(116)	897 b	(104)	863 b	79
Total dry weight at maturity (g/m^2^)	2017	1401 ab	(108)	1273 bc	(98)	1300 abc	138
	2018	1330 abc	(123)	1199 cd	(111)	1079 d	
	2019	1431 a	(117)	1295 abc	(106)	1223 c	
	Average	1387 a	(116)	1256 b	(105)	1200 b	80
Harvest index of total brown rice (%)	2017	35.6 d	(89)	38.2 bc	(96)	40.0 a	1.4
	2018	37.2 bc	(94)	38.6 ab	(97)	39.7 a	
	2019	35.6 d	(94)	36.9 cd	(98)	37.8 bc	
	Average	36.2 c	(92)	37.9 b	(97)	39.2 a	0.8
Harvest index of brown rice with thickness ≥ 1.5 mm (%)	2017	34.9 d	(90)	37.3 bc	(96)	39.0 a	1.4
	2018	36.2 cd	(94)	37.1 bc	(97)	38.4 ab	
	2019	35.0 d	(95)	36.2 cd	(98)	37.1 bc	
	Average	35.6 c	(93)	37.1 b	(97)	38.4 a	0.8

Values followed by the same letter within each trait are not significantly different at the 5% level of probability, determined by LSDs in the table. ( ): Percentage of 5867-36 or Koshi-36 to IR36.

**Table 3.** Dry weight of total brown rice, increase of total dry weight after 80%-heading and amount of translocation in 5867-36, Koshi-36 and IR36 from 2017 to 2019

Traits	Years	5867-36		Koshi-36		IR36	LSD (5%)
Dry weight of total brown rice ^1)^ (g/m^2^) (a)	2017	498 ab	(96)	486 ab	(94)	519 a	42
	2018	494 ab	(115)	463 bc	(108)	428 c	
	2019	510 a	(110)	478 ab	(103)	462 bc	
	Average	501 a	(107)	476 b	(101)	470 b	24
Increase of total dry weight after heading (g/m^2^) (b)	2017	347 a	(99)	330 a	(95)	349 a	118
	2018	395 a	(126)	361 a	(115)	314 a	
	2019	413 a	(118)	386 a	(111)	350 a	
	Average	385 a	(114)	359 a	(106)	338 a	68
Amount of translocation (g/m^2^) (c)	2017	151 a	(89)	156 a	(92)	171 a	108
	2018	99 a	(87)	102 a	(89)	114 a	
	2019	97 a	(87)	91 a	(81)	112 a	
	Average	116 a	(87)	116 a	(88)	132 a	63
Contribution of translocation (c/a, %)	2017	30.6 a	(93)	32.9 a	(100)	32.7 a	23.1
	2018	20.1 a	(76)	22.0 a	(83)	26.6 a	
	2019	18.8 a	(77)	19.2 a	(79)	24.4 a	
	Average	23.1 a	(83)	24.7 a	(88)	27.9 a	13.3

Values followed by the same letter within each trait are not significantly different at the 5% level of probability, determined by LSDs in the table. ( ): Percentage of 5867-36 or Koshi-36 to IR36. Note: c = a – b. b = ΔW. ^1)^ Estimated from the total brown rice yield containing 15% moisture (Rana *et al.* 2021).

**Table 4.** Lengths and widths of 1st to 3rd leaves, LAI and panicle number per m^2^ in 5867-36, Koshi-36 and IR36 from 2017 to 2019

Traits	Years	5867-36		Koshi-36		IR36	LSD (5%)
1st leaf length (cm)	2017	24.1 bc	(111)	23.2 bc	(107)	21.7 cd	2.7
	2018	28.5 a	(121)	22.2 bcd	(94)	23.5 bc	
	2019	24.8 b	(133)	19.9 de	(107)	18.7 e	
	Average	25.8 a	(121)	21.8 b	(102)	21.3 b	1.6
2nd leaf length (cm)	2017	42.9 b	(121)	40.1 cd	(113)	35.4 e	2.7
	2018	48.4 a	(129)	40.1 c	(107)	37.4 de	
	2019	44.4 b	(139)	36.5 e	(114)	32.0 f	
	Average	45.2 a	(129)	38.9 b	(111)	34.9 c	1.6
3rd leaf length (cm)	2017	46.1 a	(125)	43.0 c	(116)	37.0 e	2.0
	2018	46.3 a	(132)	43.9 bc	(126)	35.0 f	
	2019	45.7 ab	(129)	40.9 d	(115)	35.5 ef	
	Average	46.0 a	(128)	42.6 b	(119)	35.8 c	1.1
1st leaf width (cm)	2017	1.19 c	(100)	1.17 cd	(98)	1.20 bc	0.03
	2018	1.23 ab	(103)	1.23 a	(103)	1.20 c	
	2019	1.20 bc	(99)	1.15 d	(95)	1.20 abc	
	Average	1.21 a	(101)	1.18 b	(99)	1.20 ab	0.02
2nd leaf width (cm)	2017	0.93 bc	(96)	0.92 c	(96)	0.96 a	0.03
	2018	0.90 c	(97)	0.93 bc	(100)	0.93 bc	
	2019	0.95 ab	(100)	0.91 c	(96)	0.95 ab	
	Average	0.93 b	(98)	0.92 b	(97)	0.94 a	0.02
3rd leaf width (cm)	2017	0.78 d	(94)	0.78 d	(94)	0.83 a	0.03
	2018	0.78 cd	(95)	0.79 bcd	(96)	0.83 ab	
	2019	0.80 abcd	(98)	0.77 d	(95)	0.82 abc	
	Average	0.79 b	(96)	0.78 b	(95)	0.82 a	0.02
LAI	2017	3.40 ab	(91)	3.48 ab	(93)	3.73 a	0.70
	2018	3.25 ab	(111)	3.12 ab	(106)	2.94 b	
	2019	3.65 a	(102)	3.58 ab	(100)	3.57 ab	
	Average	3.44 a	(101)	3.39 a	(99)	3.41 a	0.40
Estimated LAI^1)^	2017	3.69 ab	(95)	3.93 a	(101)	3.87 a	0.37
	2018	3.34 bc	(106)	3.30 c	(105)	3.14 c	
	2019	3.81 a	(115)	3.39 bc	(102)	3.31 c	
	Average	3.61 a	(105)	3.54 a	(103)	3.44 a	0.22
Panicles/m^2^ ^2)^	2017	353 c	(83)	403 ab	(94)	428 a	32.0
	2018	291 d	(85)	332 c	(97)	343 c	
	2019	351 c	(87)	387 b	(96)	404 ab	
	Average	332 c	(85)	374 b	(95)	392 a	18.5

Values followed by the same letter within each trait are not significantly different at the 5% level, determined by LSDs in the table. ( ): Percentage of 5867-36 or Koshi-36 to IR36. ^1)^ The length × width values from 1st to 3rd leaves in a culm were cumulated, and this cumulated value (m^2^) was multiplied by panicle number per m^2^. ^2)^ Quated from Rana *et al.* (2021).

**Table 5.** ANOVA for leaf angles (degrees) of 5867-36, Koshi-36 and IR36 at each of the four mesuring times in 2017 and 2018, in which the results are shown by F-values

No. of days after 80%-heading	Lines-variety (L)	Years (Y)	Interaction (L × Y)
5 dayas	13.4**	25.6**	11.7**
15 days	13.4**	98.0**	4.9*
25 days	9.2**	102.0**	3.4
Maturity	5.7*	110.8**	0.2

Degrees of freedom for the lines-variety, the years, the interaction, and the error are 2, 1, 2 and 12, respectively. *, ** Significant at the 5 and 1% levels of probability, respectively.

**Table 6.** The 1st (flag)-leaf angles (degrees) of 5867-36, Koshi-36 and IR36 at the four mesuring times in 2017 and 2018

No. of days after 80%-heading	Years	5867-36		Koshi-36		IR36		LSD (5%)^1)^
5 dayas	2017	4.0 a	(c)^2)^	3.3 a	(b)^2)^	3.9 a	(b)^2)^	1.8
	2018	4.5 a	(c)	0.6 b	(bc)	–1.1 b	(d)	
15 days	2017	15.0 a	(a)	8.0 b	(a)	6.9 b	(a)	3.3
	2018	2.6 c	(c)	0.7 c	(bc)	0.8 c	(c)	
25 days	2017	13.5 a	(a)	8.1 b	(a)	7.1 b	(a)	3.1
	2018	2.3 c	(c)	1.1 c	(bc)	0.6 c	(c)	
Maturity^3)^	2017	10.0 a	(b)	7.0 b	(a)	7.5 ab	(b)	2.6
	2018	2.3 c	(c)	–0.2 c	(c)	0.8 c	(c)	
(LSD (5%))^4)^		(3.2)		(3.0)		(1.3)		

Values followed by the same letter within each measuring time are not significantly different at the 5% level of probavility, determined by the LSDs at the rightmost column of the table. ^1)^ By the use of the error variance of the ANOVA at each of the four mesuring times, assuming the three replications in each of the three lines-variety in each of the two experimental years as the source of three randomized data for error variance. ^2)^ Values followed by the same letter in parentheses within each line/variety are not significantly different at the 5% level of probavility, determined by the LSD in the parenthesis of each line/variety at the lowest row of the table. ^3)^ 30, 30 and 33 days after 80%-heading in 2017, and 28, 29 and 28 days after 80% heading in 2018, respectively, in 5867-36, Koshi-36 and IR36. ^4)^ By the use of the error variance of the ANOVA witin each of the three lines-vaiety, assuming the three replications at each of the four measuring times in each of the two experimental years as the source of three randomized data for the error variance.
